# Is electrosynthesis always green and advantageous compared to traditional methods?

**DOI:** 10.1038/s41467-020-14322-z

**Published:** 2020-02-06

**Authors:** Yong Yuan, Aiwen Lei

**Affiliations:** 1 College of Chemistry and Molecular Sciences, the Institute for Advanced Studies (IAS), Wuhan University, Wuhan, 430072 P. R. China; 2 National Research Center for Carbohydrate Synthesis, Jiangxi Normal University, Nanchang, 330022 P. R. China

**Keywords:** Synthetic chemistry methodology, Electrocatalysis, Sustainability

## Abstract

While electrosynthesis represents a green and advantageous alternative to traditional synthetic methods, electrochemical reactions still suffer from some drawbacks that require further efforts in order to fully express the potential of electricity-driven transformations. In this Comment, we will briefly discuss both the advantages and limitations of electrosynthesis, especially when compared with the other traditional synthetic organic methods, and share some forward-looking thoughts on the future developments of electrochemical reactions.

Electrochemistry has a long history^1^ and its origin can be traced back to 1800, when physicist Alessandro Volta invented the first electric battery that can generate continuous current^2^. Organic electrosynthesis means synthesizing organic compounds through the use of electricity. After 200 years of development, organic electrosynthesis has attracted increasing attention of scientists^3–8^. Especially in the past decade, considerable advances on electrochemical organic synthesis have been achieved^3–8^. Usually, electrochemistry is recognized as an environmentally benign tool for organic synthesis. However, in fact, hazardous substrates or reagents, a large amount of undesired by-products, etc. are sometimes involved in electrochemical reactions due to the original design of the process itself or the need for improving the reaction efficiency. Although electrochemistry itself is a green and powerful platform for organic synthesis, this does not mean that all electrochemical reactions are environmentally more friendly compared to traditional methods. In this comment, we will briefly discuss this topic.

## Requirement for oxidants and reductants

**Fig. 1:**
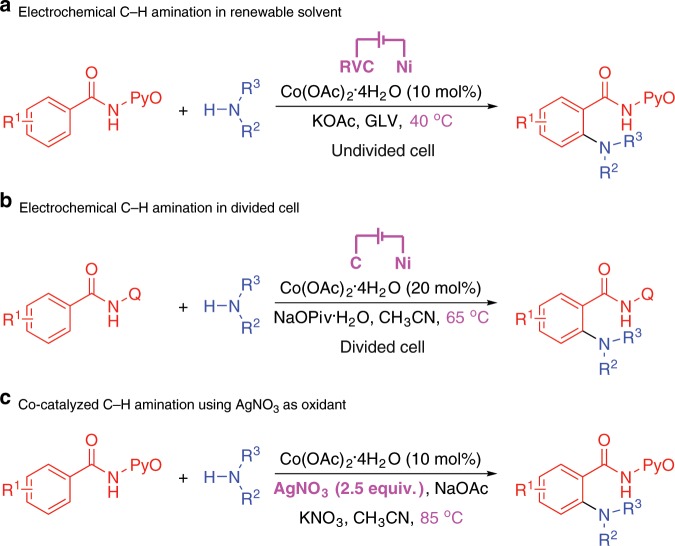
Oxidative C–H amination of aromatic amides. **a** Electrochemical C–H amination in renewable solvent. **b** Electrochemical C–H amination in divided cell. **c** Co-catalyzed C–H amination using AgNO_3_ as oxidant.

Redox reactions are of fundamental importance in chemical science and are widely investigated and applied in synthetic organic chemistry. Traditionally, in order to realize oxidation or reduction processes, stoichiometric amounts of oxidants or reductants are required. Though these approaches are reliable for producing target compounds, the use of stoichiometric amounts of oxidants or reductants inevitably leads to some hazardous waste, posing environmental concerns to the widespread application and upscaling of these methods. Organic electrosynthesis is an efficient and powerful synthetic tool, which can realize redox transformations via anodic oxidation and cathodic reduction, thereby under exogenous-oxidant-free and reductant-free conditions^3–8^. From the point of avoiding the use of stoichiometric amounts of oxidants or reductants, organic electrosynthesis is undoubtedly a much greener synthetic strategy than traditional methods. For instance, in 2018, Ackermann and co-workers reported an electrochemical C–H amination protocol using aromatic amides and cyclic secondary amines in renewable solvent (tetrahydro-2H-pyran-2-one, Fig. 1a)^9^. Almost at the same time, the Lei group reported a Co-catalyzed electrochemical C–H amination reaction between aromatic amides and secondary amines in a divided cell (Fig. 1b)^10^. Compared to the traditional method that employs 2.5 equiv. of AgNO_3_ as the oxidant (Fig. 1c)^11^, the cobalt catalyst was directly recycled by anodic oxidation in these two electrochemical approaches, thus avoiding the generation of waste products caused by oxidants and offering a cleaner C–H amination protocol.

## Waste prevention

**Fig. 2:**
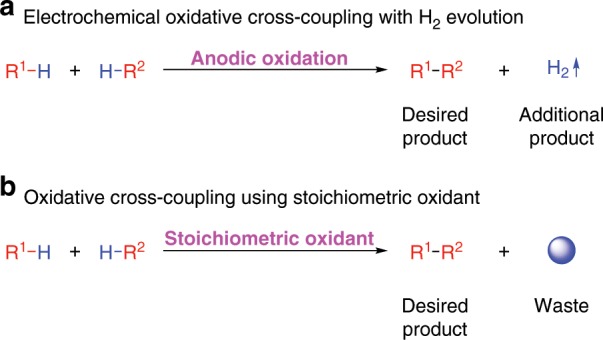
Oxidative cross-coupling reactions of R^1^-H with R^2^-H. **a** Electrochemical oxidative cross-coupling with H_2_ evolution. **b** Oxidative cross-coupling using stoichiometric oxidant.

Along with the rapid development of industry in the modern economy model, the environmental burden of chemical production is becoming more concerning. In order to address this issue, scientists are trying to find methods to reduce or, even more desirably, avoid the generation of waste. In this regard, chemists are paying attention to developing reactions that take place under waste-free conditions. Electrochemical oxidative cross-coupling of R^1^-H with R^2^-H is a promising and green method for bond formation^12^, which can not only access the desired cross-coupling product via direct or indirect anodic oxidation under metal or metal-free conditions, but also generates valuable hydrogen gas as additional product via the concomitant cathodic protons reduction (Fig. 2a). Compared with the traditional oxidative cross-coupling methods that generate a large amount of waste in most cases (Fig. 2b), electrochemical oxidative cross-coupling of R^1^-H with R^2^-H leads to the same product under waste-free conditions and is undoubtedly much greener.

## More advantages of electrosynthesis compared to traditional methods

Being environmentally friendly is just one of the advantages of electrosynthesis. From the viewpoint of practical operation, there are several incentives that should encourage chemists to use organic electrosynthesis. For example, under exogenous-oxidant-free or exogenous-reductant-free conditions, electrochemical reactions usually exhibit good functional group tolerance. Compared to traditional methods that often proceed at elevated temperature or pressure, electrochemical reactions are usually carried out under milder conditions providing an energy-saving option. Reaction times for electrochemical reactions are usually short due to their high reaction efficiency. Moreover, by increasing the operating current, the reaction time can be further shortened. By varying the current or voltage, the oxidation or reduction capacity of the electrochemical system can be optionally altered, thereby achieving the reactions that cannot occur with chemical oxidants or reductants. Compared to traditional reactions that frequently need to be quenched, electrochemical reactions can be easily stopped at any time by turning off the power switch. Finally, most electrochemical reactions are easily scaled up and have a great potential in industrial applications. For example, the electrochemical synthetic methods for 1,4-dicyanobutane, sebacic acid, etc. have successfully achieved the industrialization^13^.

## Not all electrochemical reactions are green

Green Chemistry is defined as the “design of chemical products and processes to reduce or eliminate the use and generation of hazardous substances”^14^. According to the 12 Principles of Green Chemistry^15^, there are several criteria to assess whether a reaction is green or not or to compare the environmental impact of different reactions by looking at specific metrics. Avoiding the use of stoichiometric amounts of oxidants/reductants and/or producing valuable hydrogen gas are not a guarantee that electrochemical reactions are green. Taking into account the whole set of Green Chemistry principles allows for a complete assessment of the environmental impact of a chemical transformation. For example, hazardous electrolytes are employed in several organic electrosynthesis, as beneficial additive for improving conductivity in solution, however this results in a higher explosion risk. Toxic, flammable, or corrosive solvents may also be chosen for certain electrochemical reactions impacting negatively the safety features of the overall process. Environmentally unfriendly additives may also be involved in electrosynthesis and contribute to the production of large amounts of waste. In all the above cases, electro-organic synthesis fails to comply with crucial guidelines of Green Chemistry.

## Practical limitations of electrosynthesis

Despite having many advantages, drawbacks still exist in the implementation of electrosynthesis in organic chemistry. For example, a complete electrochemical device is necessary, and its price and maintenance are usually not cheap. Furthermore, in order to promote the electron transfer in solution, a supporting electrolyte usually has to be employed and since tetrahydrofuran, toluene, etc. are poorly conductive solvents, the choice for solvent is sometimes difficult in electrosynthesis. Since most metal cations are easily reduced at the cathode to zero-valent metals, the use of metal catalysts under easily available undivided cell is relatively limited in electrochemical reactions and when electrochemical reactions are performed in divided cell, expensive ion exchange membranes are necessary for dividing anode and cathode.

## Conclusions and outlook

In summary, organic electrosynthesis can realize selective redox transformations under exogenous-oxidant-free and exogenous-reductant-free conditions through electron transfer on the electrode surface. Looking forward, in order to develop more efficient and green electrochemical synthetic methods, perhaps organic chemists should focus on the following aspects in the future: (1) using organic electrochemistry to deal with the challenges that cannot be solved by traditional methods; (2) developing new electrochemical mediators, electrode materials, electrolytic cells, and ion exchange membranes; (3) minimizing, recycling, and even avoiding the use of supporting electrolytes; (4) asymmetric electrochemical transformations are still less developed, thus we should pay more attention to the development of enantioselective electrochemical reactions; (5) compared to the extensive study of electrochemical oxidation reactions, relatively less examples on electrochemical reduction reactions are reported until now and it would be worthwhile to pay more attention to cathodic reactions in the future; (6) in most reported reactions, either anodic oxidation or cathodic reduction leads to the desired products, however, some sacrificial reagents are often needed to promote the reactions that take place at counter electrode, like protic solvents, metal electrodes, etc. To deal with this problem, we may pay more attention to the paired electrolysis that combines anodic oxidation and cathodic reduction synergistically leading to the target products. (7) Finally, the design and manufacture of novel electrolyzers, such as continuous-flow electro-reactor should aim to make electrosynthesis more efficient.
